# Study on the Dynamic Characteristics of Low-Frequency High-Stiffness Viscoelastic Damping Structures

**DOI:** 10.3390/ma18071446

**Published:** 2025-03-25

**Authors:** Zhangda Zhao, Wenjun Meng, Bijuan Yan, Huijun Liang, Yihang Geng, Zhengyu Sun

**Affiliations:** 1 School of Mechanical Engineering, Taiyuan University of Science and Technology, No. 66, Waliu Road, Wanbailin District, Taiyuan 030024, Chinas202312210068@stu.tyust.edu.cn (Y.G.);; 2 Key Lab for Optoelectronic Technology and Systems, Ministry of Education, College of Optoelectronic Engineering, Chongqing University, Chongqing 400044, China

**Keywords:** viscoelastic suspension, vibration-damping structure, elastic modulus, dynamic mechanical performance, stiffness-damping characteristics

## Abstract

Viscoelastic suspension systems serve as components essential for enhancing ride comfort and structural reliability in tracked bulldozers. However, traditional suspension-damping structures often exhibit high-stiffness and reduced-damping characteristics under low-frequency excitations, limiting their adaptability in complex off-road conditions. This limitation adversely affects the operational safety and comfort of tracked bulldozers, particularly in extreme terrains. This study employs an elastic modulus gradient design, combined with the hysteretic properties of viscoelastic materials, aiming to develop five rubber materials with varying elastic moduli and constitutive parameters for damping-layer applications. Through parametric modeling and finite-element simulations, we systematically analyzed both static and dynamic performance across these five configurations while investigating how damping-layer elastic modulus variations influence dynamic vibration-damping characteristics. The results demonstrate that the S-NR3-NR3-S configuration exhibits superior dynamic vibration-damping performance. Further analysis focused on this optimal configuration’s stiffness characteristics and energy dissipation capacity under different harmonic excitations and maximum compression displacements. Finally, practical validation was conducted through static and dynamic performance testing of physical prototypes using a universal testing machine and electro-hydraulic servo-controlled instrumentation, based on operational conditions of high-power tracked bulldozers. The presented findings and methodologies establish a theoretical foundation for optimizing ride comfort in construction vehicles, demonstrating significant practical applicability and engineering value.

## 1. Introduction

In recent years, the frequent occurrence of natural disasters has resulted in the need for construction vehicles to have excellent safety and high reliability [1]. In this context, construction vehicles must operate in complex and harsh environments, such as rugged off-road terrain and extreme weather conditions, which places higher demands on their suspension system’s dynamic performance [2]. Viscoelastic suspension systems play a crucial role in improving vehicle stability, ensuring mission success and safeguarding operator safety, owing to their superior damping ability and durability. Therefore, after in-depth studies, the dynamic response characteristics and reliability of viscoelastic suspensions under extreme conditions have become the core technologies used to enhance the performance of emergency rescue vehicles.

To ensure the operational safety and reliability of construction vehicles, passive constrained damping structures are typically used in viscoelastic suspensions. Common passive constrained damping structures include free-constrained damping structures [3], sandwich damping structures [4,5], and adjustable viscoelastic damping [6]. In industrial applications, rubber-based sandwich damping structures are widely used due to their excellent damping performance, energy absorption properties, low cost, and high mechanical reliability. Examples include the viscoelastic dampers in high-speed military tracked vehicles [7], viscoelastic suspensions in hill-climbing off-road vehicles [8], nonlinear rubber springs in railway vehicles [9], vibration isolation systems in naval ship containers [10,11] and engine mounts, and tractor cabin isolation systems [12]. To characterize the nonlinear mechanical vibration-related properties of rubber-based damping structures, Sjoberg and Kari [13] experimentally investigated the effects of nonlinear excitation on the dynamic stiffness and damping of rubber dampers, finding that the stiffness of rubber dampers exhibits high nonlinearity, and that damping is influenced by the amplitude of harmonic excitation. Banic et al. [14] used numerical simulations to study the heat generation in rubber dampers under cyclic loads and employed the Bergstrom–Boyce viscoelastic constitutive model to predict hysteretic behavior and heat generation. Huang et al. [15] combined numerical and experimental analyses to investigate the dynamic properties of viscoelastic damping isolators under impact loads. Meran et al. [16] simulated the damping performance of automotive suspension elastomer dampers under different excitation frequencies using numerical methods.

These studies indicate that the viscoelastic material in the damping suspension or structure largely determines the damping characteristics and energy dissipation efficiency of the system. Therefore, exploring and characterizing the dynamic properties and damping performance of intermediate viscoelastic materials has become a critical aspect of the design of efficient damping structures. However, most existing studies focus on static material properties, with a lack of comprehensive understanding of dynamic responses. Furthermore, viscoelastic materials exhibit significant behavior differences under varying operational conditions, making further research on their performance under complex loading conditions essential, along with the development of accurate mechanical constitutive models.

Rubber-based polymers, or rubber materials, are commonly selected as viscoelastic materials in suspension systems, as they exhibit both superelastic and viscoelastic characteristics, showing large deformation, nonlinearity, incompressibility, and variable stiffness. Under dynamic excitation, rubber materials can convert part of the mechanical energy into thermal energy, exhibiting hysteretic effects and energy dissipation capabilities which result in vibration damping and shock resistance [17,18]. Viscoelastic suspensions take advantage of the composite properties of rubber polymer materials, which combine elastic solids and viscous liquids, and display three main characteristics under mechanical loading: creep, stress relaxation, and hysteretic response [19]. Creep refers to the slow deformation of the material under a constant load over time; stress relaxation refers to the gradual decrease in stress under constant strain; and hysteretic response is characterized by a lag between the loading and unloading paths in the stress–strain curve, with the area between these paths representing the energy dissipated by the viscoelastic material [20,21]. Therefore, the elastic properties of viscoelastic materials can store strain energy under small deformations, while providing the stiffness necessary for the dynamic response process, ensuring that the viscoelastic suspension can return to its normal state during operation.

Since linear viscoelastic theory tends to underestimate the magnitude of hysteresis loops under large strains, leading to errors in energy dissipation predictions, superelastic, linear, and viscoelastic models should be used in hysteresis analysis [22]. The stress response in a super-viscoelastic model consists of an instantaneous nonlinear elastic part and a time-delayed viscous part. Previous studies have also analyzed the behavior of viscoelastic materials, considering lumped spring-damping systems. For example, Bhave et al. analyzed the energy dissipation and nonlinear viscoelastic behavior of tire rubber under friction on rough surfaces and developed a corresponding analytical model [23]. Park et al. proposed a bilinear dual-objective model used to calculate the dynamic parameters of structures and validated the hysteretic response of high-damping laminated rubber bearings through cyclic shear tests [24]. Sun et al. explored the stiffness-damping characteristics of viscoelastic suspensions, using a negative stiffness approach [25]. However, lumped spring-damping systems have limitations in representing rubber materials, especially as to the nonlinear large-deformation characteristics exhibited by rubber polymers. Dynamic simulation calculations often experience convergence issues, and discrepancies exist between material properties and actual definitions, leading to high uncertainties in dynamic testing, especially under dynamic-impact and high-load conditions in viscoelastic suspensions. Therefore, it is essential to develop constitutive models that accurately represent the nonlinear characteristics of rubber polymers and properly capture their mechanical properties.

To address these issues, this study proposes a fractional-order constitutive model-based fast-solving method to accurately and efficiently characterize the nonlinear behaviors of damping layers under wide range of strain-rate conditions. This model overcomes the limitations of traditional viscoelastic models by providing a more precise theoretical framework for analyzing dynamic mechanical properties, particularly under extreme working conditions. Complementing the modeling effort, this study systematically investigates the structural mechanics-related performance of damping layers with different elastic moduli, using an elastic modulus gradient concept. Five viscoelastic suspension damping structures are developed, and their static and dynamic performance is analyzed using the Mooney–Rivlin superelastic model and the Prony series viscoelastic model through APDL simulation. The impact of the damping layer’s elastic modulus on key performance metrics, such as static stiffness, dynamic stiffness, and loss factors, is clarified.

Furthermore, a composite rubber viscoelastic damping structure is proposed, integrating experimental validation and finite-element simulations. The experimental results, obtained under the practical operating conditions of a tracked bulldozer, are consistent with simulation trends, validating the model’s effectiveness. The combined modeling and experimental approach not only elucidates the relationships between material properties and structural responses but also provides actionable insights for future designs, such as gradient-transition-layer structures. These contributions lay a solid theoretical foundation for understanding and optimizing the performance of advanced damping structures while enhancing the safety and operational comfort of tracked vehicle systems.

The structure of this paper is organized as follows: Section 2 presents five design schemes for a viscoelastic suspension damping structure and identifies the constitutive parameters governing each material layer. Section 3 comparatively analyzes the static/dynamic mechanical properties of various suspension configurations through experimental testing and finite-element simulations, with particular emphasis on the frequency-dependent dynamic response behavior of the S-NR3-NR3-S architecture. Section 4 synthesizes the key findings of this study.

## 2. Structural Design

### 2.1. Structural Model

**Figure 1:**
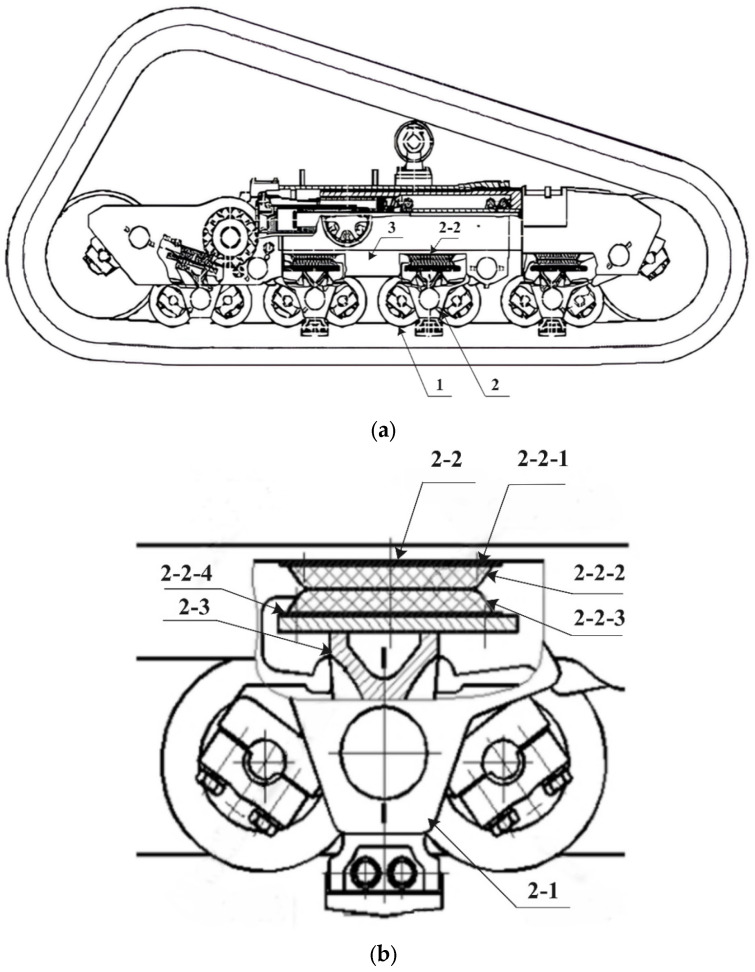
Schematic diagram of the installation of the K-type viscoelastic suspension. (**a**) Schematic diagram of crawler walking system with a viscoelastic suspension damping structure. (**b**) K-type viscoelastic suspension. 1: Roller; 2: K-type viscoelastic suspension; 2-1: swing arm; 2-2: viscoelastic damping structure, 2-2-1: upper supporting steel plate, 2-2-2: upper damping layer; 2-2-3: lower damping layer; 2-2-4: lower supporting steel plate; 2-3: intermediate reinforcement; 3: bench frame.

This paper focuses on the “K”-type viscoelastic suspension device as the object of investigation (see Figure 1a). The viscoelastic damping structure (VDS) of the suspension exhibits excellent damping effects and high energy dissipation capacity, providing superior vibration-damping performance, strong adjustability, impact resistance, and the ability to withstand harsh operating conditions. Figure 1b presents the 2D dimensional diagram of the K-type viscoelastic suspension, which consists of a swing arm, viscoelastic damping structure, spring damper, limiting device, and bench frame.

As shown in Figure 1b, the viscoelastic damping structure 2-2 consists of supporting steel plates and viscoelastic damping elements. It includes two free-constrained damping structures, referred to as the upper damping structure and the lower damping structure, which are vertically aligned and assumed to experience only normal separation and friction. The upper and lower parts are composed of constraint layer steel plates and viscoelastic rubber layers (2-2-1 and 2-2-2 form the upper damping structure, while 2-2-3 and 2-2-4 form the lower damping structure). Both structures share identical geometric dimensions and bonding methods and are integrated using vulcanization and mechanical connections.

**Figure 2:**
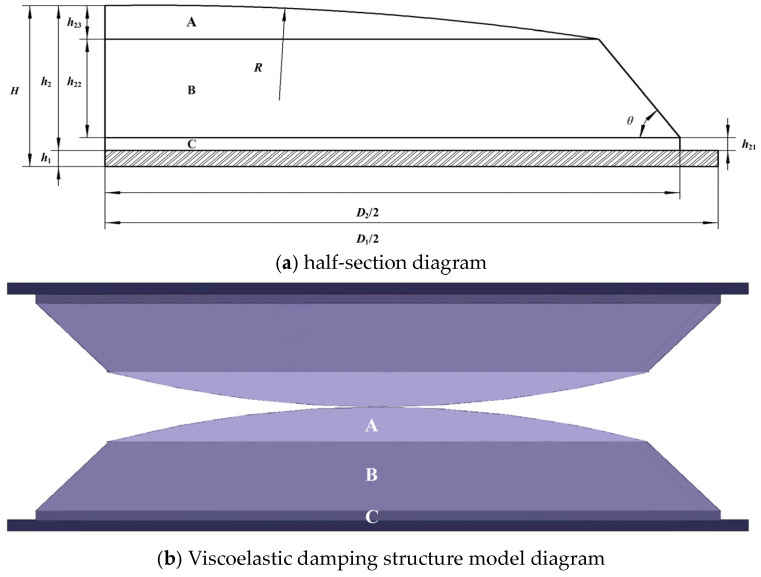
Viscoelastic damping structure.

Figure 2a shows a half-sectional view of the viscoelastic damping structure. *D*_1_ represents the diameter of the supporting steel plate, and *D*_2_ represents the bottom diameter of the viscoelastic damping element. *H* is the total thickness of one side of the damping structure, which is determined by the maximum compression displacement under different working conditions. *R* represents the radius of the dome-shaped area in the damping layer (Region A), *θ* represents the inclination angle of the side surface of Region B, *h*_21_ is the thickness of Region C, *h*_22_ is the thickness of Region B, and *h*_23_ is the thickness of Region A. The maximum compression displacement is typically set at 20% of the total thickness of the damping rubber layer, as exceeding this value may lead to stress fatigue and affect the dynamic performance. If the compression displacement is less than 20%, the damping effect becomes less significant. The 20% compression displacement is considered the threshold for both safety margin and optimal damping performance. The *h*_1_ represents the thickness of the constrained layer supporting steel plate, and *h*_2_ represents the thickness of the damping-layer viscoelastic damping element.

Based on past engineering design experience, the damping layer is divided into three functional regions: A, B, and C. Region A is the viscoelastic energy-dissipating zone (See Figure 2b), which has a dome-shaped structure located at the top of the damping element. When subjected to ground excitation or vehicle body vibrations, the rubber in region A of the upper and lower damping structures undergoes contact, compression, friction, and collision, resulting in frictional energy dissipation at the rubber’s surface and molecular chain breakage and reorganization inside the rubber, converting mechanical energy into heat energy. Region B is the support and vibration-damping zone, which has a frustum shape located in the middle of the damping element. When the rubber in region A is compressed, region B supports it, maintaining the energy dissipation and stiffness stability, while also playing a rebound damping role, particularly under extreme working conditions in which large compression displacements occur. Region C is the transition connection zone, cylindrical in shape, located at the bottom of the damping element. Region C serves as the connection with the supporting steel plate and acts as a transition zone for excitation transmission, helping to buffer the transmission of external forces or vibration waves.

To ensure the reliability of the damping-layer rubber and its superior energy-dissipating damping characteristics, the thickness ratios of the three regions were determined based on the design rule that the maximum compression displacement should be 20% of the total thickness *H*, and referencing the design theory in Ref. [25]. The thickness ratios were specified as *h*_21_ = 9%, *h*_22_ = 68%, and *h*_23_ = 23% (See Table 1). This design allows the damping layer to fully exhibit its excellent energy-dissipation properties while maintaining sufficient stiffness, ensuring the safety and operational stability of the tracked walking system under complex working conditions. Based on the aforementioned design method and referencing the specific numerical parameters provided in [26], the main geometric parameters of the viscoelastic damping structure were determined, as shown in Table 1.

### 2.2. Material Properties

The properties of the viscoelastic material determine the performance of the viscoelastic damping structure and the dynamic characteristics of the suspension. Therefore, this study focuses on the impact of the elastic modulus of the damping-layer material on the structural dynamic performance, using elastic modulus and hardness as constraint conditions. Five different rubber materials were selected for the damping layer, with the structural arrangement shown in Table 2. The abbreviations for SR, SBR, NR, NBR, and FKM in Table 2 are explained in the Abbreviations section at the end of the manuscript.

**Figure 3:**
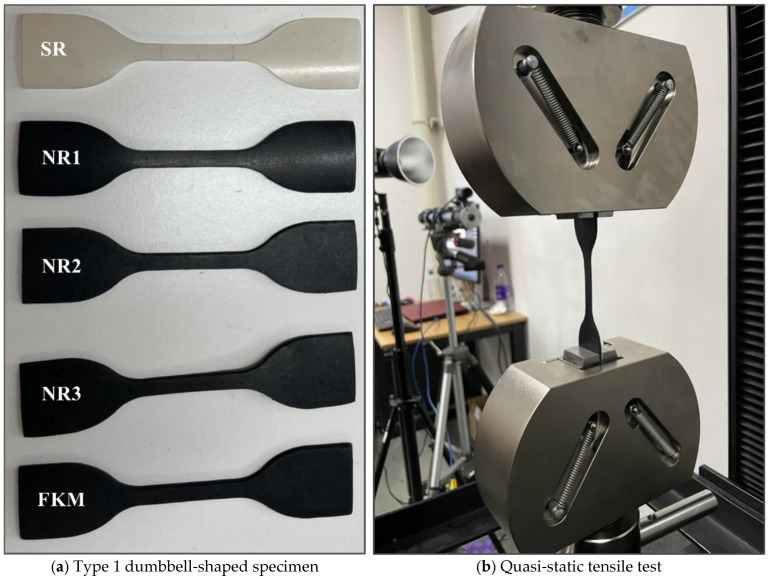
Quasi-static tensile test of rubber.

The five rubber materials listed in Table 2 were procured from Dongguan Zhisheng Rubber & Plastic Technology Co., Ltd. (Dongguan City, Guangdong Province, China). To obtain the characteristic parameters of each rubber material, quasi-static tensile tests were conducted according to the GB/T 528-2009/ISO 37:2005 standard, i.e., the “Determination of Tensile Stress–Strain Properties for Vulcanized or Thermoplastic Rubber”. Dumbbell-shaped specimens of Type 1 with a thickness of 2 mm were prepared following the standard (see Figure 3a). The tests were performed using an INSTRON 68TM-5 dual-column electronic tensile testing machine (Instron Corporation, headquartered in Boston, MA, USA) with a force measurement accuracy of 0.001 N (see Figure 3b). The tensile loading rate was set at 500 mm/min, and the specimens were stretched until failure. All tests were conducted at room temperature, with three specimens prepared for each material. The average of the three measurements was used as the final result. The hardness of the rubber materials was measured using an A-type Shore durometer manufactured in Dongguan, China; five measurements were taken for each material, and the average values are presented in Table 3.

This paper establishes a hyperelastic model for the rubber materials considered, based on the assumptions of continuum mechanics, to analyze their mechanical response under static loading. The model is based on the Mooney–Rivlin (M-R) framework, originally proposed by Mooney and later refined by Rivlin to include a generalized strain energy function. The general form of the model is expressed as

(1) $$W(I_{1},I_{2})=\sum_{i,j}^{\infty }C_{ij}{\left(I_{1}-3\right)}^{i}{\left(I_{2}-3\right)}^{j}$$

where *C_ij_* are material constants. Typically, *C*_00_ = 0.

Under uniform deformation, the relationship between stress and strain can be expressed as

(2) $$\sigma =2[{\left(1+\varepsilon \right)}^{2}-{\left(1+\varepsilon \right)}^{-1}][\frac{\partial W}{\partial I_{1}}+{\left(1+\varepsilon \right)}^{-1}\frac{\partial W}{\partial I_{2}}]$$

Here, *I*_1_ and *I*_2_ are the first and second invariants of the deformation tensor.

To simplify the calculations, researchers introduced a two-parameter Mooney–Rivlin model (MR(2)), expressed as

(3) $$W=C_{10}(I_{1}-3)+C_{01}\left(I_{2}-3\right)$$

where *C*_10_ and *C*_01_ are material constants.

In the process of tensile or compressive deformation, an important descriptive parameter is the stretch ratio, denoted as *λ*, which is defined as

λ=1+ΔL/L=1+ε

For unconfined deformation, $\lambda_{1}=\lambda ,\lambda_{2}=\lambda_{3}=\lambda^{-\frac{1}{2}}$.

The uniaxial stress expression is then given by

(4) $$\sigma =\left[C_{10}\left(\lambda -\frac{1}{\lambda^{2}}\right)+C_{01}\left(\frac{1}{\lambda }-\frac{1}{\lambda^{2}}\right)\right]$$

For uniaxial tension, the first and second invariants are expressed as

(5) $$\left\{\begin{array}{l} I_{1}=2\lambda^{-1}+\lambda^{2}=2{\left(1+\varepsilon \right)}^{-1}+{\left(1+\varepsilon \right)}^{2}\\ I_{2}=2\lambda +\lambda^{-2}=2(1+\varepsilon )+{\left(1+\varepsilon \right)}^{-2} \end{array}\right.$$

By substituting *I*_1_ and *I*_2_ into the stress equation, the uniaxial stress expression simplifies to

(6) $$\sigma =2[{\left(1+\varepsilon \right)}^{2}-{\left(1+\varepsilon \right)}^{-1}][C_{10}+C_{01}{\left(1+\varepsilon \right)}^{-1}]$$

**Figure 4:**
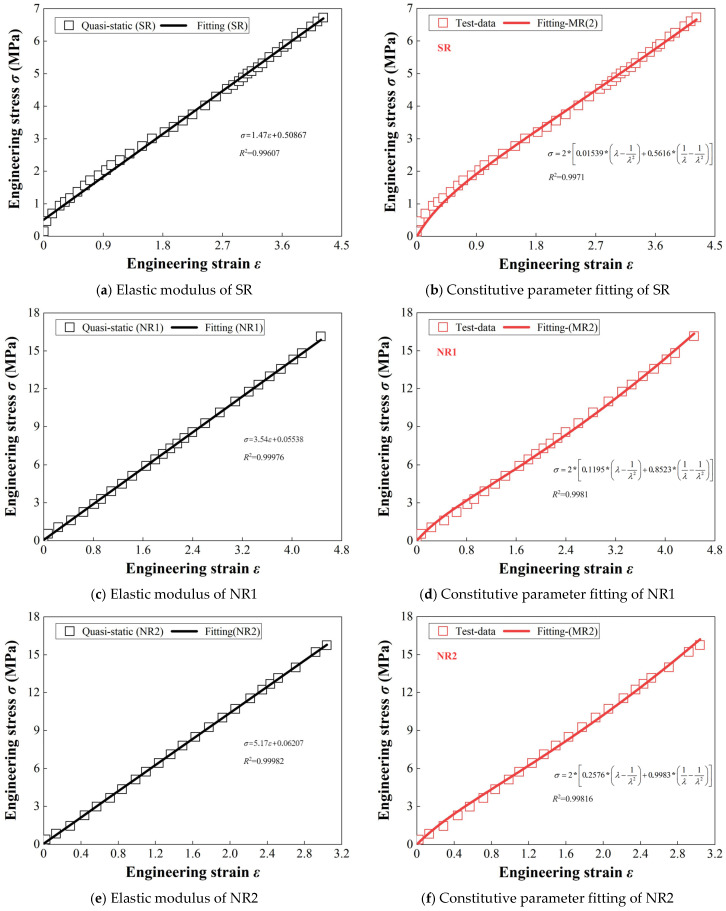

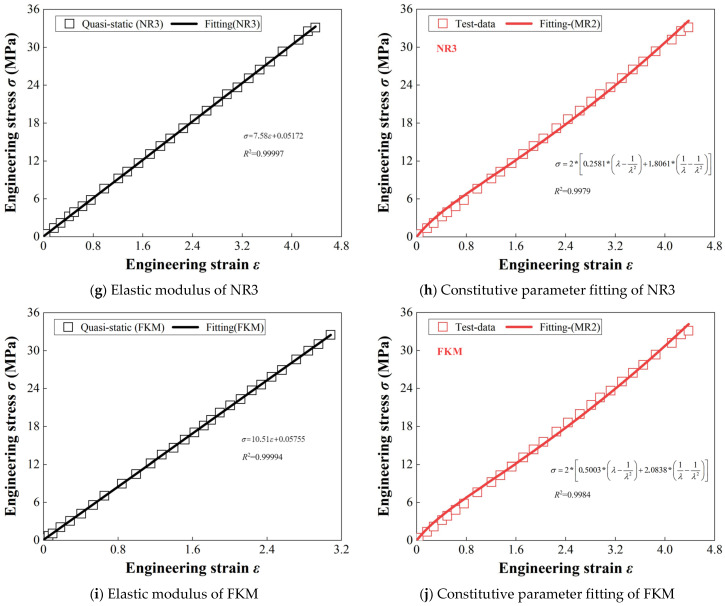
Fitting curves of elastic moduli and constitutive parameters of the MR (2) model for five types of rubber.

Using the stress–strain data obtained from the experiments, the elastic moduli and Mooney–Rivlin constitutive parameters of the five rubber materials were calculated and fitted. The fitting results are shown in Figure 4. From Figure 4a,c,e,g,i, it can be observed that fluoroelastomer (FKM) has the largest elastic modulus, followed by NR3, NR2, and NR1, with styrene-butadiene rubber (SR) showing the smallest elastic modulus. The elastic moduli and stress–strain curves of NR3, NR2, and NR1 differ due to their different filler formulations. NR1 contains 20% styrene–butadiene rubber (SBR), which has better wear resistance. Although its strength is lower than that of natural rubber (NR), its hardness and elastic modulus are slightly higher than NR, leading to higher rigidity under both static and dynamic loading. NR2 contains nitrile rubber (NBR), and the presence of the nitrile group increases the polarity of NBR, enhancing the molecular interactions within the rubber.

As a result, NR2 exhibits a higher elastic modulus and rigidity compared to NR, with an elastic modulus typically ranging from 2 to 10 MPa, depending on the nitrile content and formulation. Therefore, NR2’s elastic modulus is greater than that of NR1. NR3 contains fluoroelastomer (FKM), which has a high crosslinking density and rigid molecular structure, resulting in high polarity and rigidity, making it particularly suitable for high-temperature and highly corrosive environments. FKM’s hardness and elastic modulus are among the highest in the rubber industry. The addition of FKM to NR aims to improve its rigidity, high-temperature resistance, and weather resistance, enhancing the reliability of the VDS in complex working conditions, while ensuring safe rigidity and strength alongside its excellent energy dissipation characteristics.

This paper uses the Mooney–Rivlin two-parameter model to fit the quasi-static stress–strain characteristics of five types of rubber, with the fitting results shown in Figure 4b,d,f,h,j; the constant terms of the fitting formulas in each figure correspond to *C*_10_ and *C*_01_ in Table 3. In the figures, the fitting correlation *R*^2^ is used to represent the accuracy of the model in fitting the mechanical properties of each rubber material. It can be observed that the *R*^2^ values for all five rubber materials exceed 99%, indicating that the Mooney–Rivlin two-parameter model can accurately describe the quasi-static mechanical behavior of these rubber materials. The constitutive parameters for each rubber are listed in Table 3.

The large deformation issue in suspension damping structures primarily involves the damping layer. While traditional integer-order hyperelastic or viscoelastic models offer high computational efficiency, they often oversimplify the nonlinear and time-dependent behaviors of materials under conditions of large deformation, leading to discrepancies with real working conditions.

To address this issue, this study introduces the Viscoelastic Fractional Kelvin–Voigt (VFKV) model, developed based on our previous research. By incorporating fractional-order derivatives, this model can more accurately capture the nonlinear stress–strain relationships, strain-rate dependency, and time-dependent viscoelastic effects of materials under conditions of large deformation. The key features and derivation process of the model are outlined below.

**Figure 5:**
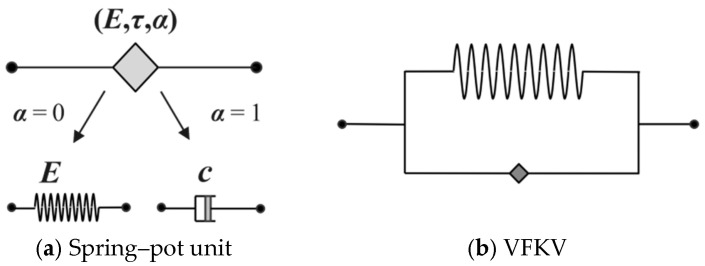
Schematic diagram of the fractional derivative basic unit model and the fractional derivative Kelvin–Vogt model.

As shown in Figure 5, the spring–pot unit is now commonly used to describe the complex nonlinear behavior of stress and strain instead of the spring and dashpot units. The constitutive relationship of this unit not only simplifies the number of parameters of the model but is also more applicable. Its fractional-order derivative constitutive relationship is

(7) $$\sigma \left(t\right)=E\tau^{\alpha }\frac{d^{\alpha }\varepsilon \left(t\right)}{dt^{\alpha }},0\leq \alpha \leq 1$$

where *E* is the elastic modulus of the material, *τ* is the relaxation time (constant), *t* is the loading time, *ε*(*t*) denotes the strain, *σ*(*t*) denotes the stress, and *α* represents the order of the fractional-order derivative model for viscoelastic unit, which is able to describe the evolution trend of the curve of the complex mechanical properties of the rubber in the process of deformation in time, and it is valued in the range of 0 to 1. When *α* = 0, the viscoelastic unit transforms into a linear spring unit, and when *α* = 1, then it corresponds to a dashpot unit.

According to the Boltzmann superposition principle, the stress response of Equation (7) can be transformed into

(8) $$\sigma \left(t\right)=E\tau^{\alpha }\Gamma \left(1-\alpha \right)D^{\alpha }\varepsilon \left(t\right),0\leq \alpha \leq 1$$

where *D^α^* represents the fractional derivative operator and Γ(·) denotes the gamma function. Because the viscoelastic response parameters of the spring–dashpot unit are time- and strain-rate-dependent, the transformation equation, Equation (9), can be obtained.

(9) $$\sigma (\varepsilon )=\frac{E{\left(\dot{\varepsilon }\tau \right)}^{\alpha }\varepsilon^{\left(1-\alpha \right)}}{\Gamma (2-\alpha )},0\leq \alpha \leq 1$$

Equation (9) represents the constitutive model referred to as the fractional-order Boltzmann model.

Figure 5b shows the physical form of the VFKV constitutive model, which is represented by parallel connection of the spring–pot units and the spring units. The integer-order constant term dashpot *c* shown in the figure is converted into a viscoelastic function model containing (*E*, *τ*, *α*). Considering that the fractional-order parameter *α* exhibits a power-law relationship with material properties, the VFKV model can be expressed as follows:

(10) $$\sigma =E\varepsilon +c{\dot{\varepsilon }}^{\alpha (\varepsilon )}\frac{\varepsilon^{\left(1-\alpha (\varepsilon )\right)}}{\Gamma (2-\alpha (\varepsilon ))},\alpha (\varepsilon )=A\varepsilon^{B}+Z$$

where coefficients *A*, index *B*, and parameter *Z* are constants.

**Figure 6:**
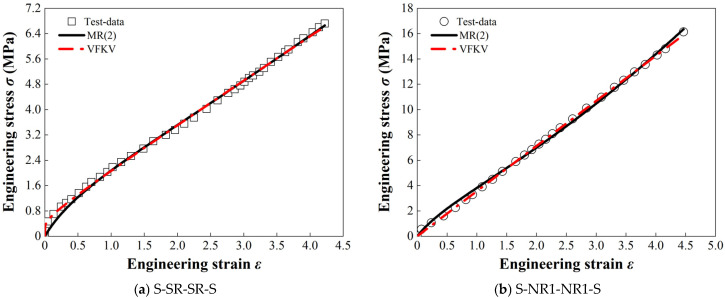

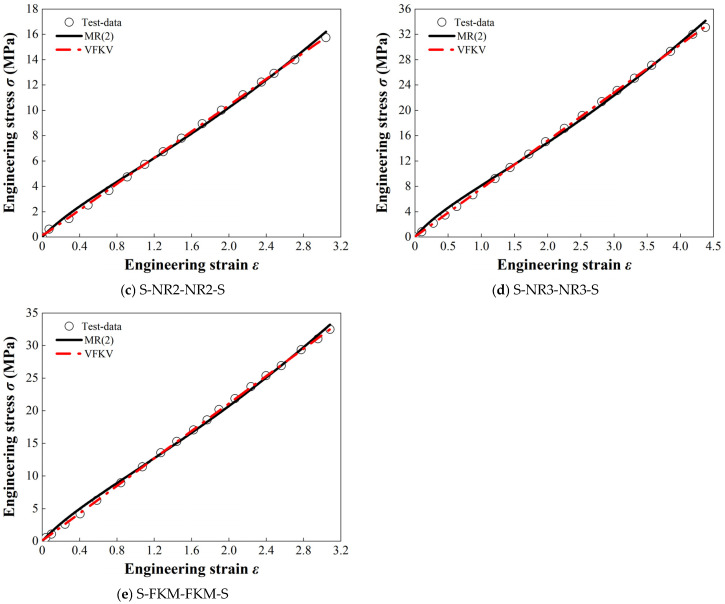
Comparison of the fitting curves of the VFKV model and the MR(2) model for the mechanical responses of different rubber materials.

One of the significant advantages of the VFKV model is that its fractional order *α* demonstrates a power-law relationship with material properties, enabling real-time characterization of the large-deformation response of materials. As shown in Figure 6, the VFKV model and the MR(2) model were both used to fit the stress–strain curves of the structure. The results clearly indicate that the VFKV model provides a more accurate fit for the quasi-static mechanical responses of the various rubber materials, compared to MR(2).

Figure 6 underscores the significant advantages of the VFKV model over the MR(2) model in modeling the mechanical responses of various rubber materials. The VFKV model excels in both small and large-deformation regions, demonstrating superior accuracy and adaptability across different materials. For instance, in Figure 6a, corresponding to the S-SR-SR-S structure, the VFKV model (red curve) demonstrates exceptional accuracy in capturing the nonlinear behavior in the initial small-deformation region. In the context of suspension system engineering applications, small strain is typically defined as a deformation of less than 5% of the total damping-layer thickness, corresponding to the linear or quasi-linear elastic response of the material.

In Figure 6b,c, for the S-NR1-NR1-S and S-NR2-NR2-S structures, both models provide reasonable fits across the medium-strain range, which ranges from 5% to 15% of the total damping-layer thickness. This range represents the onset of nonlinear behavior, in which the stress–strain relationship begins to deviate significantly from linearity, and energy dissipation becomes more pronounced. However, the VFKV model outperforms MR(2) in terms of precision, particularly under conditions of nonlinear stress–strain evolution. In Figure 6d, corresponding to the S-NR3-NR3-S structure, the superiority of the VFKV model becomes more evident under high-stress conditions, a term which encompasses large strains exceeding 15% of the total damping-layer thickness. In this regime, viscoelastic materials exhibit fully nonlinear and large-deformation characteristics, accompanied by significant energy dissipation. The VFKV model consistently aligns with experimental data, capturing the complex stress–strain responses of viscoelastic materials more accurately than the MR(2) model.

### 2.3. Mechanical Properties of the Damping Layer

In engineering applications, rubber is chosen as a damping and energy dissipation material due to its unique molecular chain structure and dynamic mechanical properties. The molecular chains of rubber are long-chain structures capable of undergoing significant sliding and bending under external forces, exhibiting pronounced viscoelastic characteristics. Particularly under alternating stresses, when the molecular chain movement cannot promptly respond to the loading rate, internal friction occurs. This is accompanied by sliding and entanglement of the molecular chains, leading to a hysteretic effect. This effect causes part of the input energy to dissipate as heat, converting mechanical energy into thermal energy. When stress and strain are used to characterize the behavior of molecular chains under alternating stress, a phase difference *φ* between the two variables can be observed in the stress–strain curve during dynamic cyclic loading. The mathematical expression for this process is as follows:

(11) $$\varepsilon =\varepsilon_{0}e^{iwt},\sigma =\sigma_{0}e^{i(wt+\varphi )}$$

where *σ*_0_ represents the initial alternating stress value, *ε*_0_ represents the initial alternating strain value, *w* is the excitation frequency, *t* is the loading time, and *φ* is the phase angle.

For the VDS of the viscoelastic suspension in this study, the energy dissipation and vibration reduction are achieved through the hysteretic effect of the damping-layer rubber. Therefore, in exploring the vibration-damping performance of the viscoelastic suspension, it is essential to first clarify the energy-dissipating capacity of the damping-layer rubber component. When rubber materials are subjected to sinusoidal excitation, the constitutive equation that includes their hysteretic characteristics can be expressed as

(12) $$\sigma =E^{\prime }(1+i\eta )\varepsilon$$

where $E^{\prime }$ is the storage modulus, and *η* is the loss factor, $\eta =E^{\prime \prime }/E^{\prime }$.

The magnitude of the phase angle *φ* indicates the degree to which stress lags behind strain, reflecting the energy dissipation-related characteristics of the material. Additionally, *η* = tan*φ*, which implies *φ* = arctan(*η*). By combining Equations (11) and (12), Equation (13) can be obtained.

(13) $$\sigma =\sigma_{0}\cos(wt+\varphi )=E^{\prime }\varepsilon_{0}\sin(wt)+\eta E^{\prime }\varepsilon_{0}\cos(wt)=\sigma_{e}+\sigma_{d}$$

where *σ*_e_ represents the elastic portion of the stress *σ*, and $\sigma_{e}=E^{\prime }\varepsilon_{0}\sin(wt)$; *σ*_d_ represents the energy-dissipating portion of the stress *σ*, and $\sigma_{d}=\eta E^{\prime }\varepsilon_{0}\cos(wt)$.

To obtain the hysteresis loop of the rubber material, *σ*_d_ can be further expressed as

(14) $$\sigma_{d}=\eta E^{\prime }\varepsilon_{0}\cos(wt)=\pm \eta E^{\prime }\sqrt{\varepsilon_{0}^{2}-\varepsilon_{0}^{2}\sin^{2}(wt)}=\pm \eta E^{\prime }\sqrt{\varepsilon_{0}^{2}-\varepsilon^{2}}$$

Rearranging Equation (14), we obtain the following:

(15) $${\left(\sigma_{d}/\eta E^{\prime }\right)}^{2}+\varepsilon^{2}=\varepsilon_{0}^{2}$$

**Figure 7:**
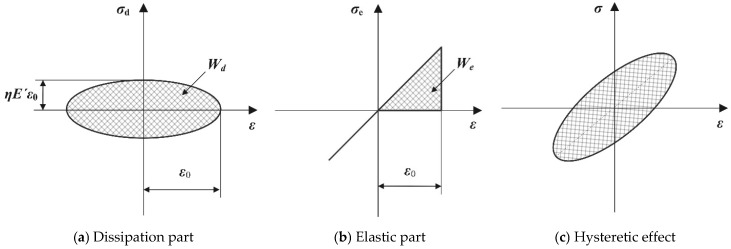
Hysteretic effect of rubber material.

Based on Equations (13)–(15), the hysteresis loop of the rubber element is obtained as shown in Figure 7. The area enclosed by the curve in Figure 7a represents the energy dissipated by the rubber during cyclic deformation, which shows the relationship between dissipated stress (*σ*_d_) and strain. Figure 7b corresponds to the elastic stress component (*σ*_e_) and its relationship with strain, reflecting the stress response of the rubber under elastic deformation. Figure 7c combines the curves from Figure 7a,b, providing the total stress–strain relationship for the rubber material, as described in Equation (11).

In Figure 7a, *W_d_* represents the energy dissipated by the rubber material during a complete vibration cycle, and its expression is

(16) $$W_{d}=\int_{0}^{\frac{2\pi }{w}}\sigma_{d}\frac{d\varepsilon }{dt}dt=\pi \eta E^{\prime }\varepsilon_{0}^{2}$$

In Figure 7b, *W_e_* only shows the energy of one-quarter of the vibration cycle. Combining this with Equation (12), the loss factor *η* can be determined as:

(17) $$\eta =\frac{W_{d}}{2\pi W_{e}}$$

Equation (17) and Figure 7 both clearly illustrate the physical significance of the loss factor, which is the ratio of the energy dissipated by the material to the energy stored.

In addition, the horizontal axis in Figure 7 represents strain, while the vertical axis represents stress. This conversion is based on the relationship between force and displacement; thus, the data obtained from the experiment need to be appropriately converted. Let us assume that at any given time *t*, the excitation applied to the viscoelastic damping structure is

(18) $$F=m\ddot{x}(t)$$

At the same time *t*, the compression displacement of the 2-2-3 is

(19) $$x=x_{1}(t)-x_{2}(t)$$

where *x*_1_(*t*) and *x*_2_(*t*) represent the input and output displacements at time *t*, respectively.

Therefore, the constitutive equation defining the relationship between the excitation force and the displacement of the damping layer can be expressed as

(20) $${\left(\frac{F-kx}{\eta kx_{0}}\right)}^{2}+{\left(\frac{x}{x_{0}}\right)}^{2}=1$$

In the equation, *x*_0_ represents the maximum compressive displacement of the damping layer.

## 3. Dynamic Characteristics Analysis of VDS

Given the balance between computational efficiency and accuracy, as well as the minimal impact of the steel frame structure on the damping performance of the K-type viscoelastic suspension, this study focuses exclusively on investigating the dynamic characteristics of the viscoelastic damping structure while ensuring the validity of the analysis results. Based on the geometric parameters outlined in Table 1 of Section 2.1, a finite-element model of the viscoelastic suspension was established, and its damping performance was analyzed using ANSYS APDL 18.2 software.

During modeling, contact pairs have been defined between layers in the ANSYS APDL model. The contact interface between the steel plates and the damping layer in Region C is defined as bonded (always) contact to simulate the vulcanized adhesion observed in real-world applications. For the internal regions of the damping layer, bonded (always) contact is applied, ensuring that the damping layer is treated as a unified structure during the analysis. To simulate the dynamic contact state of the rubber components, rough contact pairs were established at the interfaces of the A regions between the upper and lower damping layers. Considering the stick–slip friction behavior observed at the contact surface between the two rubber blocks, the friction coefficient between the A regions of the two damping layers is set to 0.2.

**Figure 8:**
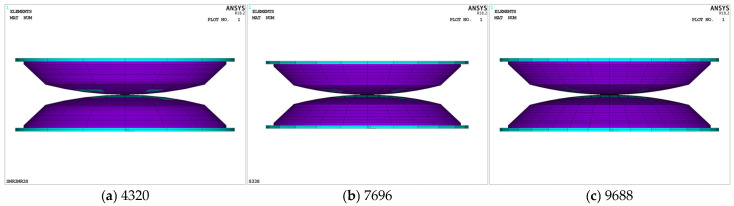
Model diagrams for three different mesh element counts.

Considering the importance of mesh element accuracy and uniformity in achieving reliable computational results, especially given the large-deformation nonlinear characteristics of rubber, a refined local quarter model was adopted to evaluate the effects of three different mesh refinement levels on iteration count and solution accuracy (see Figure 8). The findings indicated that a mesh with 7696 elements (see Figure 8b and convergence curves under static and transient dynamics) provided the optimal balance between computational accuracy and efficiency. Iterations stabilized effectively, demonstrating good convergence properties, and the results were consistent with those obtained from a model with 9688 elements. However, excessive mesh refinement (as shown in Figure 8c) not only leads to significant deformation of the damping layer, but also exacerbates the computational efficiency of the overall structure.

The supporting steel plates of the viscoelastic damping structure were modeled using the Solid185 element, while the damping layer was modeled with the Solid186 element. The material properties for both elements were defined according to the parameters listed in Table 3. To simulate the nonlinear characteristics of the damping-layer rubber material, this study not only fitted the two-parameter Mooney–Rivlin (MR) hyperelastic model for the rubber, as shown in Table 3, but also calculated the third-order Prony series of the rubber based on relaxation and creep tests. The specific values are provided in Table 4.

While the MR model is commonly used for hyperelastic materials due to its ability to capture static and quasi-static behavior, it has limitations in accurately modeling the dynamic performance of damping rubber under high-frequency loading. Comparative analyses have demonstrated that the VFKV model offers superior fitting accuracy for dynamic properties, particularly in capturing the frequency-dependent damping characteristics of rubber. However, the current study utilized a combination of the Prony series and the MR model because the VFKV model has not yet been fully implemented in the simulation software used. This combined approach was chosen as a practical compromise to incorporate both the hyperelastic behavior described by the MR model and the viscoelastic time-dependent characteristics captured by the Prony series, ensuring a more comprehensive representation of the material’s behavior under static and dynamic loading conditions.

We recognize the potential benefits of using the VFKV model for improved dynamic fitting and plan to implement it in future studies through secondary software development. The decision to use the combined MR and Prony series model in this study reflects the current computational constraints and the need to balance accuracy with practicality. This approach effectively captures the key dynamic and nonlinear characteristics of the damping layer while maintaining compatibility with existing simulation tools. By laying the groundwork with this hybrid model, we aim to refine the methodology further once the VFKV model is successfully integrated into the simulation framework.

### 3.1. Static Analysis

**Figure 9:**
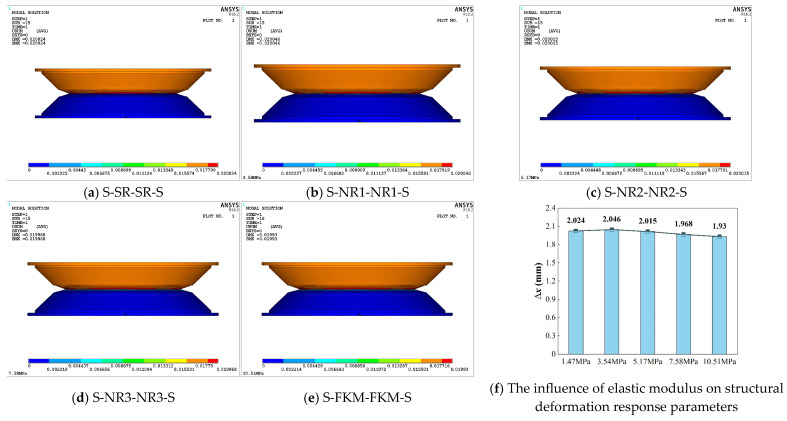
The influence of damping layers with different elastic moduli on structural deformation response parameters.

The static compression displacement contour plots of the five structures are shown in Figure 9, illustrating the deformation at the final loading sub-step for each structure. It can be observed that no element failure occurred in any of the structures, and all the maximum displacement values exceeded the applied loading amplitudes, a finding which can primarily be attributed to the nonlinear large-deformation effects of the damping-layer rubber. Among them, the S-NR1-NR1-S structure (Figure 9b, with *E*_2_ = 3.54 MPa) exhibited the largest deformation displacement, of 20.046 mm, followed by the S-SR-SR-S structure (Figure 9a, with *E*_2_ = 1.47 MPa) at 20.024 mm. The S-NR2-NR2-S and S-NR3-NR3-S structures showed displacements of 20.015 mm and 19.968 mm, respectively, while the S-FKM-FKM-S structure (Figure 9e, with *E*_2_ = 10.51 MPa) had the smallest displacement, only 19.93 mm.

A comparative analysis of these five viscoelastic damping structures reveals that as the elastic modulus of the damping layer increases, the maximum displacement first increases and then decreases. Using ∆*x* (the difference between the maximum deformation displacement and the applied displacement) as the evaluation index, Figure 9f shows that the horizontal axis represents the damping layers with varying elastic moduli, while the vertical axis represents ∆*x*. From this, it can be seen that when *E*_2_ = 3.54 MPa, ∆*x* reaches its peak, equivalent to 11.3% of the applied displacement, indicating that this material tends to experience larger nonlinear deformation losses.

Overall, under static excitation, all five structures are within the normal rubber deformation range. To further investigate the stability of each design and the effect of the damping layer’s elastic modulus on the performance of the viscoelastic suspension, a deeper analysis involving parameters such as stress, strain, and strain rate is needed.

**Figure 10:**
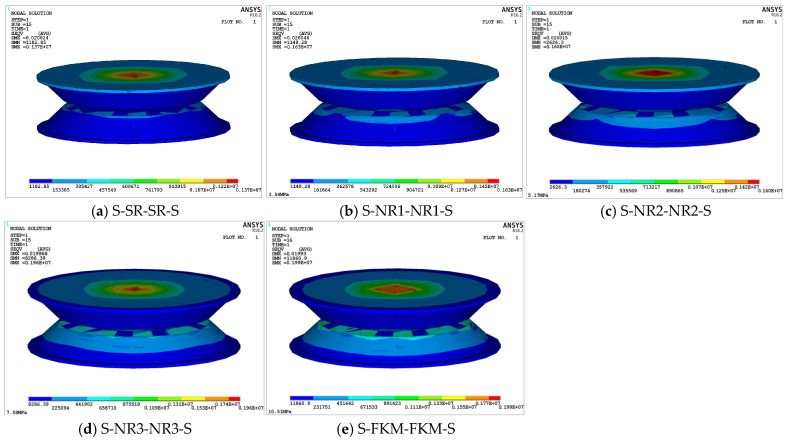
Static equivalent stress cloud maps of five VDS.

Figure 10 shows the maximum equivalent stress contour plots for the five viscoelastic damping structures. It can be observed that the maximum stress points in all structures occur at the center of the supporting steel plate 2-2-1 in the upper damping structure. Among them, the S-NR2-NR2-S structure (Figure 10c) exhibits the largest stress concentration area. In the horizontal direction, the stress gradually decreases from the center to the boundary along the radius, while in the vertical direction, the stress decreases along the concentric axis. Notably, when the elastic modulus of the damping layer exceeds 7.58 MPa (Figure 10d,e), the rate of stress expansion decreases, and the stress concentration area in the central region becomes smaller compared to the first three structures. However, the stress values in these regions are significantly larger than those in the other three structures. For example, the minimum stress for the structures with damping-layer elastic moduli of *E*_2_ = 7.58 MPa and *E*_2_ = 10.51 MPa are 3.15 times and 4.51 times greater, respectively, than the minimum stress for the *E*_2_ = 5.17 MPa structure.

Moreover, by examining the contour plots of the lower damping layer 2-2-3 in Figure 10a–e and analyzing the corresponding data, it can be observed that as the elastic modulus of the damping layer increases, the stress transfer between the layers becomes more pronounced. The loading stress gradually increases, and the force transfer from the upper damping structure to the lower damping structure becomes more significant. In Figure 10a, the S-SR-SR-S structure shows the lowest maximum equivalent stress value, 1.37 MPa, while in Figure 10e, the S-FKM-FKM-S structure exhibits the highest value, 1.99 MPa. The maximum equivalent stress values for the S-NR1-NR1-S and S-NR2-NR2-S structures in Figure 10b,c are 1.63 MPa and 1.60 MPa, respectively, with only a slight difference between them. The five structures are ranked by maximum equivalent stress as follows: 10.51 MPa > 7.58 MPa > 5.17 MPa ≈ 3.54 MPa > 1.47 MPa. A comparative analysis reveals that as the elastic modulus of the damping layer increases, the maximum stress initially increases, then decreases, and then increases again, although the magnitude of decrease is relatively small. Overall, higher elastic moduli enhance the maximum equivalent stress of the structure, accelerate load transfer between the layers, and increase the load-bearing capacity.

**Figure 11:**
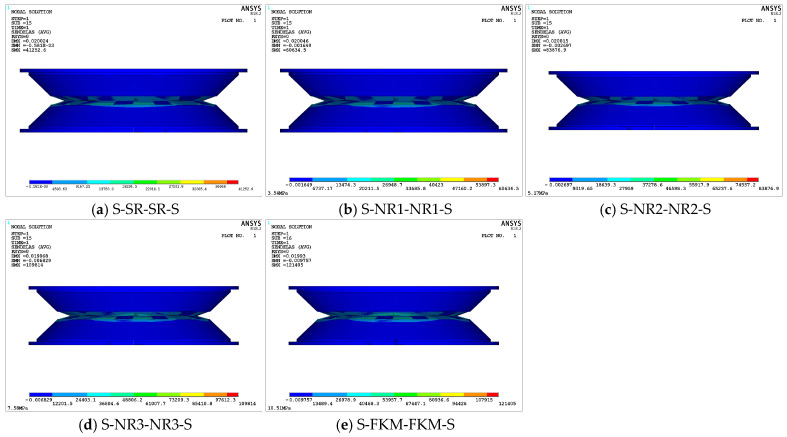
Unit strain energy density cloud maps of five VDSs.

To ensure the stability of the viscoelastic damping structure, it is essential to obtain the unit strain energy density contour plots, which help to identify high-strain regions and assess whether the maximum strain energy density falls within the allowable limits, thus preventing permanent damage to the rubber and failure of the structure. Figure 11 presents the unit strain energy density contour plots for the five viscoelastic damping structures. The highest strain energy density is concentrated in the contact region of the upper and lower damping layers and increases with the elastic modulus. Additionally, the maximum stored energy at this node follows the same ranking as the elastic moduli of the damping-layer rubber, with the S-FKM-FKM-S structure exhibiting the highest strain energy density and elastic stored energy, while the S-SR-SR-S structure shows the lowest.

Furthermore, since the peak release of unit strain energy density in each structure occurs at the rubber layer, a trapezoidal integration method, combined with the data from the five rubber models, was used to calculate the quasi-static tensile test data, in order to determine the ultimate strain energy density (*U_limit_*) for each rubber material. This allows for further assessment of whether the maximum strain energy density values are within the allowable range. The specific comparison values are presented in Table 5.

As shown in Table 5, the damping layers of all five structures fall within the allowable range. Among them, the S-NR3-NR3-S structure exhibits a significant difference from the limit range, indicating higher safety and stability. In contrast, the S-NR2-NR2-S structure is closest to the limit strain energy density value. If this viscoelastic damping structure is chosen, optimization of its structural layout and material parameters would be necessary to ensure its performance.

**Figure 12:**
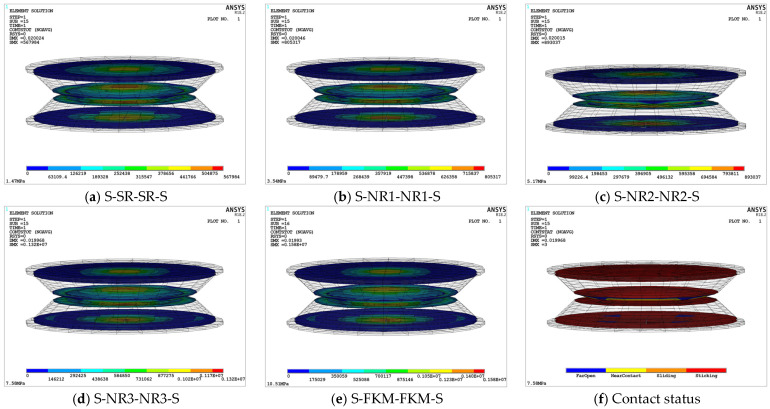
Cloud diagrams of total contact stress for five VDSs.

The viscoelastic damping structure adopts a stacked layered design, relying on the compression and friction between the upper and lower damping-layer rubber materials to convert mechanical energy into thermal energy for vibration damping. Therefore, after ensuring that the maximum unit strain energy density meets the allowable requirements, it is also necessary to consider the force transfer path, especially the force transmission between the contact surfaces. Figure 12 illustrates the stress distribution on the interlayer bonding and contact surfaces of the five viscoelastic damping structures. It can be observed that the maximum stress values of all structures occur in the central region of each layer, and the overall maximum stress values of each structure are located at the contact interface between the upper and lower damping layers. This is due to the overall design of the structure, in which the A zone of the damping layers is dome-shaped. When the upper and lower damping structures collide, the A zones of the two damping layers come into contact first, compressing each other, and the force is then transmitted bidirectionally to the B and C zones and the supporting steel plate. This transmission involves mechanical waves, including both reflected and transmitted waves.

As shown in Figure 12, the maximum total contact stress of the five viscoelastic damping structures increases with the elasticity modulus of the damping layer. The sequence of increasing stress is as follows: S-FKM-FKM-S > S-NR3-NR3-S > S-NR2-NR2-S > S-NR1-NR1-S > S-SR-SR-S. Specifically, the maximum total contact stress of S-FKM-FKM-S is 1.58 MPa, while the maximum contact stress of S-SR-SR-S is 0.5679 MPa, a difference of 2.78-fold. This is because the higher the elasticity modulus of the damping-layer rubber, the greater its hardness and stiffness, which in turn increases the contact stress. Figure 12f shows the contact state of the S-FKM-FKM-S structure, in which the overall contact surface between the supporting steel plate and the damping layer, as well as the contact center between the upper and lower damping layers, is in an adhesive state with no relative sliding.

Comparing the deformation displacement, stress, strain energy density, and contact stress data of various damping structures reveals a positive correlation between the elasticity modulus of the damping-layer rubber and the overall static mechanical performance parameters of the structure. Specifically, as the elasticity modulus increases, the values of the mechanical parameters also increase. However, excessively high mechanical parameters may increase the structure’s stiffness, thereby affecting the energy dissipation capacity. Thus, the elasticity modulus of the damping layer needs to be carefully selected. A detailed local analysis was performed to explore the performance of the upper and lower damping layers and their impact on the damping characteristics. From the overall perspective, the mechanical deformation cloud maps of all of the structures exhibit common characteristics. Therefore, after evaluating the maximum values of various parameters, the S-NR3-NR3-S structure with an elasticity modulus of 7.58 MPa is chosen as a representative example to demonstrate its static performance.

**Figure 13:**
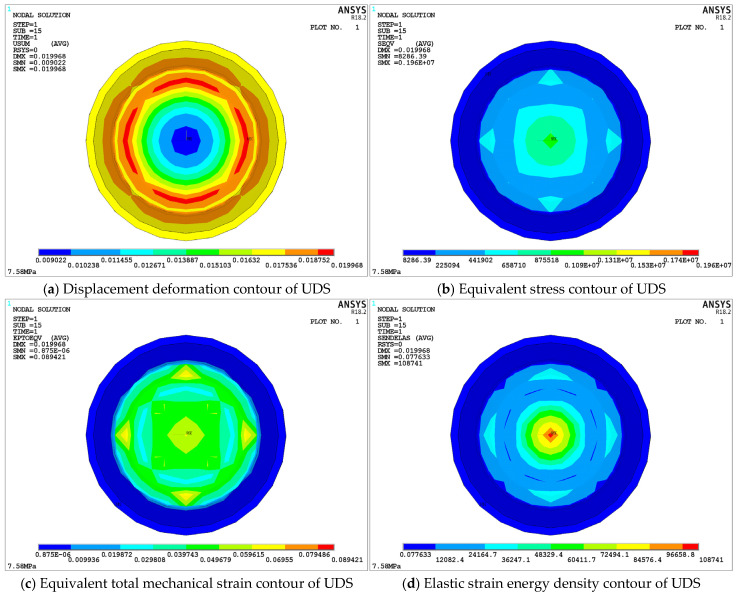
Changes in the static parameters of the UDS.

Figure 13 illustrates the static performance changes of the upper damping structure of S-NR3-NR3-S. Figure 13a shows the displacement deformation cloud map of the structure, in which the maximum deformation displacement occurs at the edge of the upper damping layer A zone, with a value of 19.968 mm, resulting in a displacement difference of 1.968 mm compared to the loading displacement response. This is due to the dome-shaped design, which causes mutual compression between the middle dome regions of both layers. Figure 13b presents the equivalent stress cloud map, in which the central stress on the A zone contact surface is 0.875518 MPa, significantly lower than the maximum equivalent stress at supporting steel plate 2-2-1. Figure 13c displays the equivalent strain cloud map, showing a smaller maximum value concentrated in the inner region. The strain values at the A zone contact surface, distributed along the circumferential direction at 90 degrees, remain within a middle range and fluctuate within the allowable limits. Finally, Figure 13d depicts the elastic strain energy density cloud map, with high energy density primarily concentrated at the center of the A zone, where the maximum value reaches 108,741 J/m^3^. This value meets the *U_limit_* of NR3 and complies with the usage requirements.

**Figure 14:**
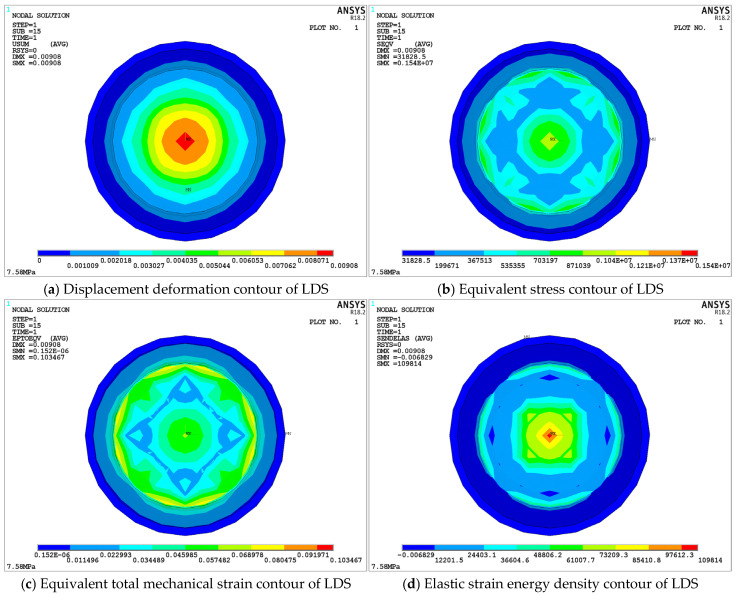
Changes in the static parameters of the LDS.

Figure 14 presents the changes in static performance parameters of the lower damping structure (LDS) of the S-NR3-NR3-S. Figure 14a shows the displacement deformation contour of the LDS. It can be observed that the maximum deformation displacement occurs at the center unit of the A zone in 2-2-3, with a value of 9.08 mm, which is approximately half of the applied displacement. This region occupies a small proportion, and the overall displacement deformation of the LDS occurs mainly in the A and B zones of 2-2-3. The displacement changes gradually in a stepwise fashion from top to bottom, with the A zone contact surface at the top (refer to the legend in Figure 14a). The displacement deformation values for the C zone and the lower supporting steel plate (2-2-4) are zero. Figure 14b presents the equivalent stress contour of the LDS. Compared to the equivalent stress in the upper damping structure (UDS), the maximum equivalent stress in 2-2-3 is smaller, with a difference of 0.42 MPa. However, the distribution trend of equivalent stress in both structures is similar and follows the same order.

Figure 14c presents the equivalent strain contour of the LDS. Similar to the UDS, the maximum equivalent strain occurs within the damping layer. The maximum equivalent strain on the contact surface of the A zone is located at the edge, distributed around 90 degrees circumferentially. Lastly, Figure 14d presents the elastic strain energy density contour of the LDS. The maximum strain energy density is located at the center node of the A zone in the 2-2-3, with a value of 109,814 J/m^3^. This is slightly larger than the corresponding value in the 2-2-2, but still much lower than the *U_limit_* for NR3, meeting the requirements for maximum compression displacement operating conditions.

By analyzing the static performance parameters of the upper and lower damping structures in the S-NR3-NR3-S configuration, it can be observed that the mechanical performance parameters of each layer are incorporated within the overall structure. Moreover, the effective contact area of the upper and lower damping layers is approximately equal to the diameter of the overlapping surface between the B and A zones. A comparison with other structures indicates that the structural performance remains within the allowable safety range. Once the consistency between the upper and lower damping layers and the overall structure’s basic mechanical performance was confirmed, the force and displacement transmission data from the top and bottom layers of each structure were further extracted (as shown in Table 6). This data was then used to investigate the force and displacement transmission rates of the damping layers, ultimately resulting in calculations of the static stiffness and static load-bearing capacity of each structure.

Table 6 presents the force and displacement amplitudes at the input and output ends of each viscoelastic damping structure. As the elastic modulus of the damping layer increases, the force transmission rate of each structure significantly increases. Among them, the force transmission rates of the S-NR3-NR3-S and S-FKM-FKM-S structures are 13.17% and 13.49%, respectively, which are notably higher than those of S-NR2-NR2-S (11.08%) and S-NR1-NR1-S (7%). The S-SR-SR-S structure is close to 0, almost achieving rigid transmission. In addition, both the load amplitude and static stiffness increase in parallel, and their ranking is consistent with the elastic modulus of the damping layer, with the highest being S-FKM-FKM-S and the lowest being S-SR-SR-S. When comparing the maximum stress and strain at the input and output ends of each structure, it is evident that both increase with the increase in the damping layer’s elastic modulus. However, the strain values are relatively small, and the effect on the deformation of the damping layer can be considered negligible.

**Figure 15:**
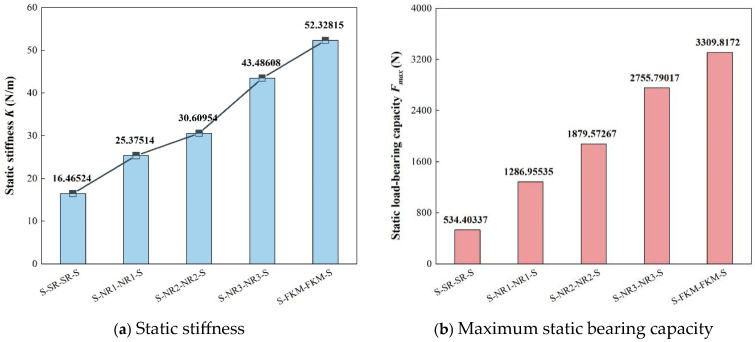
Static stiffness and static bearing capacity of five VDSs.

When evaluating the static load-bearing capacity of the structure, it is necessary to consider not only the overall load-bearing capacity but also the capacity range of the upper and lower damping layers. Based on the compressive strengths of the five types of rubber, the maximum static load-bearing capacity for each can be determined (see Figure 15b). The results show that this ranking is consistent with the elastic modulus of the damping layer: the greater the modulus, the higher the maximum static load-bearing capacity. When the elastic modulus of the damping layer is between 3.54 MPa and 7.58 MPa, the static stiffness and load-bearing capacity change significantly. When *E*_2_ ≤ 5.17 MPa and *E*_2_ = 7.58 MPa, their performance improves significantly. However, when *E*_2_ exceeds 7.58 MPa, the increase becomes slow, although its reliability still outperforms other structures.

Therefore, after a comprehensive comparison of the static performance parameters of the five VDSs, the S-NR3-NR3-S structure, with *E*_2_ = 7.58 MPa, shows the best static performance. To confirm this conclusion, further dynamic analysis will be conducted to observe its dynamic performance.

### 3.2. Sensitivity Analysis of the Elastic Modulus of the Damping Layer

Since the damping-layer material for each of the five suspension viscoelastic damping structures is selected based on the elastic modulus, the primary consideration is the influence of the damping layer’s elastic modulus on the dynamic performance of the structure. In this study, the operational conditions of a high-power (≥400 kW) crawler bulldozer are used as the loading background. According to experiments by Sun et al. [25], the primary resonance frequency range for bulldozers is 3–8 Hz, with the main resonance frequency during operation being 4.5 Hz. Therefore, this study explores the sensitivity of different rubber elastic moduli to the dynamic characteristics of the suspension at 4.5 Hz. The maximum compression displacement of the structure is used as the displacement amplitude, and uniform sinusoidal displacement excitation is applied to the upper surface of the structure. The boundary conditions are consistent with the static analysis. In addition, the complete method is used to solve for transient dynamics analysis.

**Figure 16:**
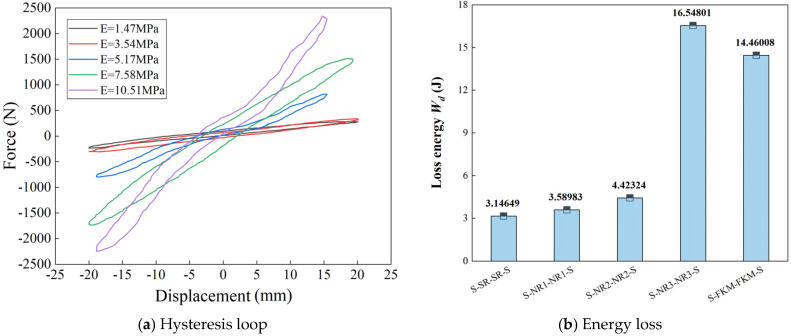
Hysteretic response for the five VDSs.

Figure 16 illustrates the relationship between the elastic modulus of the damping layer and the dynamic hysteretic response for the five damping structures. Figure 16a shows the force–displacement dynamic curves for viscoelastic damping structures with different elastic moduli. All structures exhibit hysteresis loops, indicating the energy dissipation effect of the rubber damping layer. As the elastic modulus of the damping layer increases, both the force and displacement values increase, the hysteretic response becomes more pronounced, and the dynamic energy dissipation area increases (see Figure 16b). Notably, the S-NR3-NR3-S structure exhibits the largest energy dissipation area, with a significant increase in hysteresis loop size and energy dissipation compared to the first three structures. Furthermore, the energy loss of this structure is greater than that of the S-FKM-FKM-S structure, even though the latter has a higher elastic modulus. This is because the molecular chains of FKM have higher rigidity and poor flexibility, leading to slower response and lower energy dissipation under dynamic deformation. In contrast, the weaker van der Waals forces between the molecular chains of NR make it more prone to large deformation. When blended with fluoroelastomer, the increased frequency of frictional collisions and deformation leads to greater internal energy dissipation during dynamic loading. In summary, the dynamic energy dissipation of viscoelastic damping structures is closely related to the elastic modulus of the damping layer. The damping performance of the structure is optimal when the elastic modulus is close to 7.58 MPa. Furthermore, the loss factor and stiffness values of this structure are both higher than the corresponding values reported for similar structures in Ref. [27].

### 3.3. Dynamic Characteristics of the Structure

As discussed in Section 2.3, the force–displacement hysteresis loop of the viscoelastic suspension structure characterizes its dynamic vibration-damping performance. The loop enables the determination of the structure’s dynamic load capacity, stiffness, and damping properties. To further investigate the dynamic characteristics of the viscoelastic suspension structure, harmonic transient dynamic loading was performed for the S-NR3-NR3-S structure under different alternating frequencies (3–8 Hz, the main frequency range of crawler bulldozers) and compression amplitudes. Both finite-element simulations and experimental validations were employed to evaluate the dynamic response of the structure.

**Figure 17:**
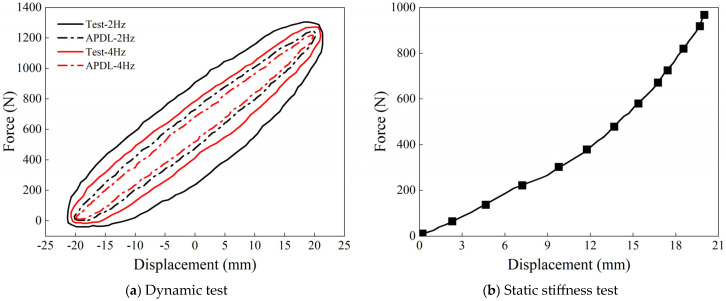
Force displacement hysteresis curves of the structure under testing and simulation at 2 Hz and 4 Hz.

In the APDL simulation, the maximum compression displacement (20% of the total structural thickness) was used as the initial displacement amplitude, and harmonic excitation was applied to the suspension structure. The damping layer was modeled using a hyper-viscoelastic constitutive model. The boundary conditions mirrored those used in static analysis, with the lower supporting steel plate fully constrained along its perimeter, the upper supporting steel plate constrained in the X-direction, and sinusoidal displacement loads applied along the Z-direction. As shown in Figure 17a, the simulated hysteresis loop responses at 2 Hz and 4 Hz indicate that the loop area decreases as the excitation frequency increases, reflecting a reduction in energy dissipation capacity. At 2 Hz, the hysteresis loop area is the largest, demonstrating the strongest energy dissipation, whereas at 4 Hz, the loop area and energy dissipation significantly decrease. The energy dissipation ratio between 2 Hz and 4 Hz is approximately 1.64 (see Table 7).

To validate the simulation results, the dynamic mechanical responses of the S-NR3-NR3-S structure were experimentally tested under low-frequency conditions (2 Hz and 4 Hz) using the WANCE HDT 255A electro-hydraulic servo fatigue testing machine (Wance Equipment Co., Ltd. in Shenzhen, China). The experimental hysteresis loops, shown in Figure 17a, exhibited significantly larger loop areas compared to the simulation results, indicating stronger energy dissipation and load-bearing capacity under actual working conditions. As summarized in Table 7, the experimentally measured dynamic properties, including static stiffness, dynamic stiffness, damping coefficients, loss factors, and energy storage capacities, were consistently higher than those obtained from the simulations. Similarly, the dynamic-to-static stiffness ratio, and loss factor errors between the two methods were 9%, and 7.7% at 2 Hz, and 9.5% and 9% at 4 Hz, respectively.

These discrepancies exceeded the typical error range of 15% associated with normal operating conditions [28,29], but remained within the allowable range of 20% associated with extreme impact scenarios [30,31,32]. Despite these numerical differences, both simulation and experimental results exhibited consistent dynamic trends: the hysteresis loop area decreased with increasing frequency, reflecting reduced energy dissipation capacity. At 2 Hz, the hysteresis loop area was the largest, demonstrating robust energy dissipation, while the displacement peak values exceeded the initial loading amplitude, highlighting the nonlinear large-deformation behavior of the rubber material under low-frequency conditions.

The observed discrepancies were primarily attributed to several factors. Firstly, the inherent nonlinearity and uncertainty of the rubber material played a significant role. Comparative analyses demonstrated that using only a hyperelastic constitutive model produced results closer to experimental values than the combined hyperelastic and Prony series model. However, the latter provided smoother calculations and better captured the dynamic and nonlinear characteristics of the damping layer, despite some deviations. This underscores the limitations of current built-in models in accurately representing the complex large-deformation behavior of rubber. Secondly, numerical errors and convergence challenges in finite-element simulations, particularly under large deformation and high-frequency loading conditions, also contributed to the differences. While finer mesh resolutions were employed to minimize numerical errors, they often led to early element failure during calculations. Simplifications in geometry and material assumptions further amplified discrepancies, as simulations tended to underestimate forces by oversimplifying a material’s nonlinear and damping effects. Lastly, the contact parameter settings significantly impacted the results. The default bonded contact parameters used in simulations inadequately captured real-world interactions, such as stick–slip friction and contact stiffness, which are critical to energy dissipation at the contact interface. Consequently, the hysteresis loop areas and dynamic responses observed in simulations underestimated the experimental results.

To address these discrepancies and improve simulation accuracy, future research will focus on the following:

(1) Optimizing Material Models: Advanced nonlinear constitutive models will be developed using user-defined subroutines (UPFs). These models will incorporate critical characteristics of rubber materials, such as strain hardening, strain softening, and frequency-dependent viscoelastic properties, to enhance the accuracy of simulations under conditions of large deformation.
(2) Improvement of Contact Models: Contact parameters, including friction coefficient, contact stiffness, and contact damping, will be refined. Experimental measurements will be conducted to validate the accuracy of contact effects in the model. This approach aims to ensure that the simulation accurately reflects the real-world interaction behavior between different structural components.
(3) Optimization of Numerical Methods: Mesh refinement will be improved to increase simulation precision, and advanced numerical integration techniques, along with adaptive time-stepping algorithms, will be adopted to reduce numerical errors in dynamic solutions. Additionally, boundary conditions will be refined to better align with real-world operating conditions, avoiding simplifications in loading paths that could lead to inaccuracies in the simulation results.

Additionally, the static stiffness data in Table 7 was obtained using the NSS CMT5605 electronic universal testing machine (Shenzhen Sansi Testing Instrument Co., Ltd. in Shenzhen, China), which was used to measure the static mechanical curve of the structure (Figure 17b). The force–displacement relationship initially exhibits linear elastic behavior. When the compression displacement exceeds 7 mm, the nonlinear effects of the rubber damping layer become dominant. Initially, as the compression displacement increases, the corresponding load grows at a slower rate, reflecting the strain-softening behavior of rubber during deformation. Once the compression displacement reaches or exceeds 15 mm, the load and material strength increase rapidly, indicating the strain-hardening behavior of the rubber.

Furthermore, the static stiffness of the structure can be derived from the mechanical response curve, as shown in Table 7. Comparing the force–displacement curves from the simulation and the experiment reveals consistent trends, but the static stiffness values differ by 4.878 N/mm. This deviation is considered normal and can be attributed to several factors, such as variations in the vulcanization process of the test specimens. In real-world tests, rubber materials exhibit true nonlinear mechanical behaviors, including strain hardening and strain softening, while the material models used in simulations are relatively simplified, often neglecting local geometric features or material inhomogeneity. Additionally, issues with the precision of mesh generation in the simulation model contribute to the discrepancies, further amplifying the differences compared to real-world conditions.

### 3.4. Mechanical Properties

From the previous analysis of experimental and simulation-based results, it is evident that both methods exhibit consistent dynamic trends: the hysteresis loop area decreases as the frequency increases, reflecting a reduction in energy dissipation capacity. At the same time, the displacement peak values exceed the loading amplitude, demonstrating the nonlinear large-deformation behavior of rubber materials at low frequencies. However, the experimental results are significantly higher than the simulation results. This discrepancy can primarily be attributed to the limitations of material models, contact parameters, and simulation methodologies.

**Figure 18:**
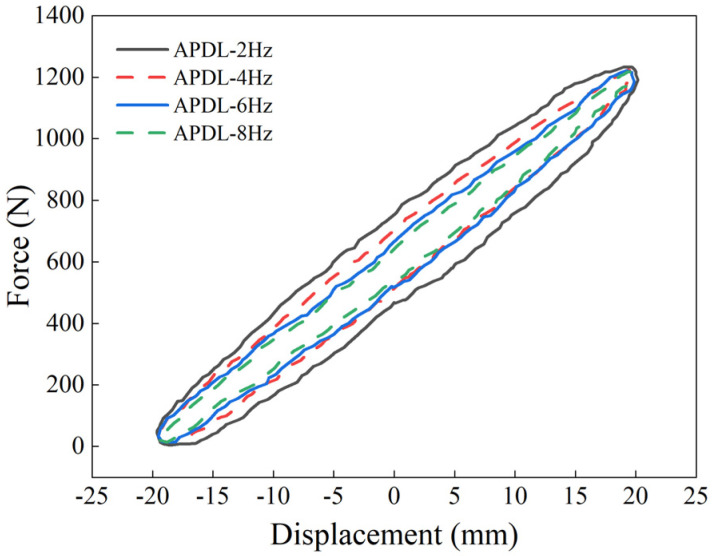
Dynamic response of VDS with different loading frequencies under maximum compression displacement.

Despite these numerical differences, simulation calculations remain advantageous in capturing trends and operational convenience. To further observe the dynamic response behavior of the structure within the main frequency range of a crawler bulldozer, the frequency range was expanded to 2 Hz, 4 Hz, 6Hz, and 8Hz, and dynamic response comparisons were conducted. As shown in Figure 18, the dynamic response of the S-NR3-NR3-S structure at these four frequencies reveals that the force–displacement hysteresis loop area decreases with increasing frequency, indicating reduced energy dissipation capacity. When the excitation frequency is 2 Hz, the force–displacement energy dissipation area is the largest, and the energy dissipation is also the highest. In contrast, at an excitation frequency of 8Hz, the energy dissipation area is smallest, and the energy dissipation is minimal. The energy dissipation ratio between these two frequency limits is 3.12.

The dynamic response curves in Figure 18 provide the stiffness and damping values of the S-NR3-NR3-S structure at different frequencies (see Table 8). The results show that as the frequency increases, the dynamic stiffness of the structure gradually increases, exhibiting a positive correlation. The dynamic stiffness is smallest at 2 Hz and largest at 8 Hz, with the rate of increase diminishing progressively. Specifically, the increase from 2 Hz to 4 Hz is 0.45 N/mm, from 4 Hz to 6 Hz is 0.25 N/mm, and from 6 Hz to 8 Hz is 0.32 N/mm. This indicates that as the frequency increases, the growth of dynamic stiffness slows down, eventually approaching a constant value. The structure becomes stiffer, but its energy dissipation capacity weakens, suggesting that the dynamic performance is better at low frequencies and that structural or material optimization is required at high frequencies.

Meanwhile, the damping coefficient decreases significantly with an increase in frequency, showing a negative correlation. This is attributed to the transition of the rubber’s nonlinear viscoelastic behavior to a more elastic response at higher frequencies, leading to reduced energy dissipation and weakened hysteretic effects. At 2 Hz, the damping coefficient is the highest, reaching 0.0275 N·s/mm, surpassing the sum of the damping coefficients at other frequencies. At 4 Hz, the damping coefficient is 0.00878 N·s/mm, which is greater than those at 6 Hz and 8 Hz, indicating that the structure exhibits superior dynamic performance at lower frequencies.

The dynamic-to-static stiffness ratio reflects the structural response under dynamic versus static loads. A high dynamic-to-static stiffness ratio indicates that the structure can maintain stable deformation under dynamic loads and withstand larger vibrations or impacts. However, if the ratio is too large, it may reduce the structure’s energy dissipation capacity. Conversely, a ratio that is too small suggests that the structure may experience excessive deformation, reduced rigidity, and a higher risk of instability or failure. In engineering applications, the typical range for the dynamic-to-static stiffness ratio is 1.4 to 1.5 [26,30]. For all four frequencies, the dynamic-to-static stiffness ratio falls within this range, and it increases with frequency. The minimum dynamic-to-static ratio occurs at 2 Hz, with a value of 1.405, and the increase in the ratio gradually decreases, ultimately stabilizing at 1.428 at 8 Hz.

The stored energy of the structure gradually decreases with increasing frequency. In contrast to the dissipated energy, the stored energy shows minimal variation between frequencies. The loss factor, which is the ratio of dissipated energy to stored energy, decreases as the frequency increases. From the data in Table 8, it can be observed that the loss factor is highest at 2 Hz, and at the resonant frequency of 4 Hz, the loss factor is approximately half of the value at 2 Hz.

In summary, it can be concluded that the S-NR3-NR3-S structure exhibits superior dynamic performance in the low-frequency range (≤4 Hz). To further investigate, the transient dynamic response of this structure at 4 Hz was examined. The response cloud diagrams provide an intuitive view of whether the structure experiences failure and show the corresponding values for displacement, stress, and strain energy.

**Figure 19:**
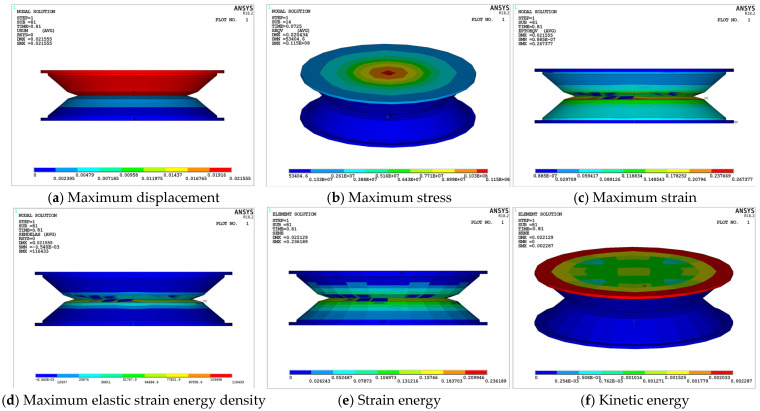
Transient dynamic response parameters of the S-NR3-NR3-S structure.

Figure 19 demonstrates that all mechanical parameters of the structure remain within safe limits, with no signs of stress concentration, strain energy concentration, or element failure. Under transient impact, the displacement amplitude of the structure exceeds the static response, and the maximum stress zone coincides with the static analysis, focusing on the central region of the 2-2-1 area. The maximum stress (Figure 19b), maximum strain (Figure 19c), and elastic strain energy density (Figure 19d) all exceed the static values. This is primarily due to the nonlinear effects of the damping layer and the inertial effects of dynamic loading, leading to substantial transient oscillations within the structure over time. The maximum stress remains within the allowable stress limits for each layer, and the region of maximum elastic strain energy density is located at the interface between the upper and lower damping layers, which is below the *U_limit_* value.

Figure 19e shows that the region with the highest strain energy is located at the contact area between the upper and lower damping layers, with a maximum strain energy of 0.236189 J. Figure 19f illustrates the distribution of kinetic energy in the structure, with the maximum kinetic energy concentrated on the 2-2-1 supporting steel plate. This is because the external excitation is directly applied to the top steel plate, generating significant vibrational energy. The top steel plate transmits kinetic energy to the lower rubber layers, which, due to their larger mass and stiffness, absorb more energy, especially in the initial stages of vibration. As the vibration propagates through the rubber layers, the kinetic energy is gradually dissipated, and the rubber layers exhibit lower kinetic energy. Given the short duration of the transient impact, the initial kinetic energy of the top steel plate is larger, with the rubber layers playing a key role in buffering and energy absorption.

**Figure 20:**
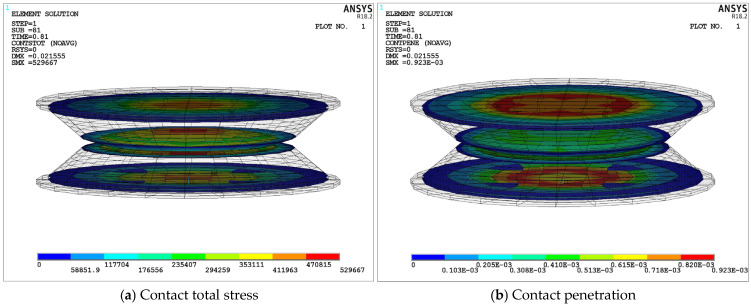
Interlayer contact state of the S-NR3-NR3-S structure.

Figure 20 further confirms the contact stability of the S-NR3-NR3-S structure under transient dynamic impacts. Figure 20a presents the total contact stress contour of the structure. From the figure, it can be seen that the maximum stress at the bonding or contact surfaces between the layers is much lower than the allowable stress of the structure. The maximum contact stress occurs at the contact surface between the upper and lower damping layers. Specifically, as shown in Figure 20b, the contact penetration status of each contact surface is presented, with the maximum penetration value being 0.923E-3mm. This indicates that there is a small overlap (less than 0.01mm) between the contact surfaces of the upper and lower damping layers. Therefore, the contact model is quite accurate, and this penetration value does not significantly affect the mechanical behavior of the structure.

Based on the results obtained from both the combined simulation calculations and experimental tests, it can be concluded that the S-NR3-NR3-S structure exhibits superior dynamic performance within the low-frequency range. Compared to other structures, it is more suitable for the operating conditions of tracked bulldozers and other construction machinery.

## 4. Conclusions

To address low-frequency, large-amplitude vibration suppression requirements in crawler suspension systems, a viscoelastic damping structure was designed through elastic modulus adjustment of the damping layer. Theoretical analysis and numerical simulations were conducted to investigate variable elasticity mechanisms by comparing static and dynamic characteristics of five VDS configurations with different elastic moduli, with analysis of how damping-layer elasticity modulus affects structural mechanical performance. The specific conclusions are as follows:

1. The mechanical performance parameters of the five designed structures are within the allowable requirements for each layer’s materials and the overall structure at maximum compression displacement. Rubber’s nonlinear effects cause maximum deformation displacement (occurring at upper/lower damping-layer interfaces) to exceed applied displacement amplitude. Structural deformation difference (Δ*x*) first increases then decreases with increasing damping-layer modulus, peaking at 11.3% of applied displacement when *E*_2_ = 3.54 MPa.
2. As the elastic modulus of the damping layer increases, the viscoelastic damping structure exhibits higher force and displacement response parameters, leading to increased static stiffness and load-bearing capacity. When *E*_2_ = 10.51 MPa, static stiffness and load-bearing capacity are both 1.2 times greater than those of the structure with *E*_2_ = 7.58 MPa, and 3.178 times and 6.19 times greater than those of the structure with *E*_2_ = 1.47 MPa. Additionally, a higher elastic modulus enhances hysteretic response, resulting in an increase followed by a decrease in dynamic energy dissipation area across structures. Notably, the S-NR3-NR3-S structure achieves the largest energy dissipation area, the energy loss value is 16.548J, surpassing the total energy dissipation of the three structures with *E*_2_ ≤ 7.58 MPa and being 1.328 times that of the *E*_2_ = 10.51 MPa structure’s energy dissipation.
3. The S-NR3-NR3-S structure demonstrates excellent dynamic damping at excitation frequencies of 4 Hz and lower. As frequency decreases, energy dissipation, damping ratio, stored energy, and loss factor increase, while dynamic stiffness rises with frequency. At 2 Hz, the loss factor peaks and nearly equals the combined loss factors at the other three frequencies. The loss factor at the primary resonant frequency of 4 Hz is about half that at 2 Hz.
4. The hysteretic effect observed in the experiment aligns with the simulations regarding frequency trends. However, the measured values for static stiffness, dynamic stiffness, damping, energy dissipation, and loss factor are significantly higher than those from simulations. For instance, at 2 Hz, the loss factor discrepancy is 0.1474–34.7% of the experimental loss factor—while at 4 Hz it rises to 58%. The errors are substantial and increase with frequency. Excluding factors like model mesh precision, material models, and contact settings reveals that the primary source of error is large deformation of the damping structure during operation. Specifically, simulation software struggles with nonlinear contact and large deformations due to (a) increased mesh precision causing non-convergence from excessive element deformation; (b) changes to layered material constitutive models leading to failure in contact surface elements; (c) inadequate ability of software modules to handle large deformations at contact interfaces and frictional energy dissipation, resulting in underestimation of damping and loss factors.

Based on the above analysis, the next steps in the research will focus on the following three areas:

(1) Developing a more refined nonlinear constitutive model for the damping layer to clarify the behavior of the material and contact interfaces under large deformations;
(2) Exploring more robust mesh generation and convergence control methods to optimize the definition and handling of contact elements;
(3) Enhancing the simulation software’s ability to represent large-deformation coupling effects, and developing more efficient algorithms or using other numerical calculation platforms to improve computational accuracy and reduce errors between simulations and experimental results.

## Figures and Tables

**Table 1:** Dimensions of the viscoelastic damping structure.

No.	Main Parameter	Value	Unit
1	Constrained Layer Diameter: *D*_1_	320	mm
2	Damping-Layer Diameter in Region C: *D*_2_	300	mm
3	Upper Damping Structure Thickness: *H*	50	mm
4	Constrained Layer Thickness: *h*_1_	5	mm
5	Damping-Layer Thickness: *h*_2_	45	mm
6	Damping-Layer Thickness in Region A: *h*_23_	10.4	mm
7	Damping-Layer Thickness in Region B: *h*_22_	30.6	mm
8	Damping-Layer Thickness in Region C: *h*_21_	4	mm
9	Dome Radius of Region A: *R*	645	mm
10	Inclination Angle of Region B: *θ*	55	°

**Table 2:** Structural design scheme.

No.	Name	Damping-Layer Material	Stacking Sequence
1	Steel-SR-SR-Steel	SR	S-SR-SR-S
2	Steel-NR1-NR1-Steel	NR1 (Containing 20%SBR)	S-NR1-NR1-S
3	Steel-NR2-NR2-Steel	NR2 (Containing 20%NBR)	S-NR2-NR2-S
4	Steel-NR3-NR3-Steel	NR3 (Containing 20%FKM)	S-NR3-NR3-S
5	Steel-FKM-FKM-Steel	FKM	S-FKM-FKM-S

**Table 3:** Material properties of components in the viscoelastic damping structure.

No.	Name	*E* (MPa)	Hardness	*Ρ* (g/cm^3^)	*ν*	Constitutive Parameters	
						*C*_10_	*C*_01_
1	SR	1.47	61HA	1.13	0.48	0.01539	0.5616
2	NR1	3.54	65HA	0.932	0.499	0.1196	0.8523
3	NR2	5.17	69HA	0.944	0.499	0.2576	0.9953
4	NR3	7.58	74HA	1.114	0.499	0.2581	1.8061
5	FKM	10.51	77HA	1.91	0.499	0.5003	2.0838
6	45#Steel	2.1 × 10^5^	197HB	7.85	0.3	\	

**Table 4:** Prony model parameters.

$\mathrm{Shear}\;\mathrm{Modulus}:G(t)=G_{\infty }+\sum_{i=1}^{N}g_{i}e^{-t/\tau_{i}}$ (MPa)	$\mathrm{Bulk}\;\mathrm{Modulus}:K(t)=K_{\infty }+\sum_{i=1}^{N}g_{i}e^{-t/\tau_{i}}$	Relaxation Time *τ*_i_ (s)
*g*_1_ = 1.02	*k*_1_ = 48.16	*τ*_1_ = 0.1
*g*_2_ = 0.612	*k*_2_ = 28.89	*τ*_2_ = 1
*g*_3_ = 0.408	*k*_3_ = 19.27	*τ*_3_ = 10

**Table 5:** Ultimate strain energy density of damping layer.

Name	Maximum Unit Strain Energy Density *U* (J/m^3^)	Ultimate Strain Energy Density of Damping Layer *U_limit_* (J/m^3^)	Permitted Status
S-SR-SR-S	41,253.1	51,057.19	Pass muster
S-NR1-NR1-S	60,635	106,400.1	Pass muster
S-NR2-NR2-S	83,878.5	84,271.42	Pass muster
S-NR3-NR3-S	109,814	243,331.8	Pass muster
S-FKM-FKM-S	121,405	167,310.5	Pass muster

**Table 6:** Force and displacement transmission data of the five VDSs.

No.	Structure Name	Layer Number	Maximum Load /N	Maximum Displacement /mm	Maximum Stress /MPa	Maximum Strain
1	S-SR-SR-S	2-2-1	329.7	18	0.37	6.53 × 10^−6^
2		2-2-4	326.9	1.05 × 10^−5^	0.354	1.69 × 10^−6^
3	S-NR1-NR1-S	2-2-1	508.67	18	1.62	7.75 × 10^−6^
4		2-2-4	472.53	1.63 × 10^−5^	0.531	2.53 × 10^−6^
5	S-NR2-NR2-S	2-2-1	612.65	18	1.6	7.63 × 10^−6^
6		2-2-4	544.73	1.72 × 10^−5^	0.568	2.71 × 10^−6^
7	S-NR3-NR3-S	2-2-1	868.33	18	1.96	9.33 × 10^−6^
8		2-2-4	753.97	2.52 × 10^−5^	0.826	3.93 × 10^−6^
9	S-FKM-FKM-S	2-2-1	1042.9	18	1.99	8.17 × 10^−6^
10		2-2-4	902.18	3.01 × 10^−5^	0.989	4.71 × 10^−6^

**Table 7:** Dynamic corresponding parameters of experiments and simulations at 2 Hz and 4 Hz.

Excitation Frequency	Condition	Static Stiffness (N/mm)	Dynamic Stiffness (N/mm)	Damping Coefficient (N·s/mm)	*W_d_* (J)	Dynamic to Static Ratio	*W_e_* (J)	*η*
2 Hz	APDL	43.486	61.09	0.0275	10.11	1.405	28.27	0.36
	Test	48.364	61.25	0.0387	14.35	1.266	36.50	0.39
4 Hz	APDL	43.486	61.54	0.00878	6.15	1.415	23.04	0.273
	Test	48.364	61.92	0.0123	10.17	1.28	32.84	0.30

**Table 8:** Stiffness damping coefficients of the S-NR3-NR3-S structure at various frequencies.

Excitation Frequency	Dynamic Stiffness (N/mm)	Damping Coefficient (N·s/mm)	*W_d_* (J)	Dynamic-to-Static Ratio	*W_e_* (J)	*η*
2 Hz	61.09	0.0275	10.11	1.405	28.27	0.36
4 Hz	61.54	0.00878	6.15	1.415	23.04	0.273
6Hz	61.79	0.00492	4.85	1.421	22.52	0.215
8Hz	62.11	0.00256	3.24	1.428	22.17	0.146

## Data Availability

The original contributions presented in this study are included in the article. Further inquiries can be directed to the corresponding authors.

## Abbreviations

VDS	Viscoelastic damping structure
LDS	Lower damping structure
UDS	Upper damping structure
SR	Silicone rubber
FKM	Fluorocarbon rubber
MR	Mooney–Rivlin
SBR	Styrene–butadiene rubber
NBR	Nitrile butadiene rubber
NR	Natural rubber
VFKV	Variable fractional-order Kelvin–Voigt constitutive model
